# Differential Activity of Plasma and Vacuolar Membrane Transporters Contributes to Genotypic Differences in Salinity Tolerance in a Halophyte Species, *Chenopodium quinoa*

**DOI:** 10.3390/ijms14059267

**Published:** 2013-04-29

**Authors:** Edgar Bonales-Alatorre, Igor Pottosin, Lana Shabala, Zhong-Hua Chen, Fanrong Zeng, Sven-Erik Jacobsen, Sergey Shabala

**Affiliations:** 1 School of Agricultural Science and Tasmanian Institute for Agriculture, University of Tasmania, Private Bag 54, Hobart, TAS 7001, Australia; E-Mails: edgarbonales@hotmail.com (E.B.-A.); L.Shabala@utas.edu.au (L.S.); Fanrong.Zeng@utas.edu.au (F.Z.); 2 University Centre for Biomedical Research, University of Colima, 28045 Colima, Mexico; E-Mail: pottosin@mail.ru; 3 School of Science and Health, University of Western Sydney, Richmond, NSW 2753, Australia; E-Mail: Z.Chen@uws.edu.au; 4 College of Agriculture and Biotechnology, Zhejiang University, Hangzhou 310058, China; 5 Department of Plant and Environmental Sciences, Faculty of Science, University of Copenhagen, Højbakkegaard Allé 13, 2630 Taastrup, Denmark; E-Mail: seja@life.ku.dk

**Keywords:** sodium exclusion, vacuolar sequestration, potassium retention, mesophyll, cytosol, H^+^-ATPase, SOS1 exchanger

## Abstract

Halophytes species can be used as a highly convenient model system to reveal key ionic and molecular mechanisms that confer salinity tolerance in plants. Earlier, we reported that quinoa (*Chenopodium quinoa* Willd.), a facultative C3 halophyte species, can efficiently control the activity of slow (SV) and fast (FV) tonoplast channels to match specific growth conditions by ensuring that most of accumulated Na^+^ is safely locked in the vacuole (Bonales-Alatorre *et al.* (2013) Plant Physiology). This work extends these finding by comparing the properties of tonoplast FV and SV channels in two quinoa genotypes contrasting in their salinity tolerance. The work is complemented by studies of the kinetics of net ion fluxes across the plasma membrane of quinoa leaf mesophyll tissue. Our results suggest that multiple mechanisms contribute towards genotypic differences in salinity tolerance in quinoa. These include: (i) a higher rate of Na^+^ exclusion from leaf mesophyll; (ii) maintenance of low cytosolic Na^+^ levels; (iii) better K^+^ retention in the leaf mesophyll; (iv) a high rate of H^+^ pumping, which increases the ability of mesophyll cells to restore their membrane potential; and (v) the ability to reduce the activity of SV and FV channels under saline conditions. These mechanisms appear to be highly orchestrated, thus enabling the remarkable overall salinity tolerance of quinoa species.

## 1. Introduction

The physiological and genetic complexity of salt tolerance significantly handicaps progress in breeding crops for this important trait [1]. Nonetheless, about 1% of land plants are not only capable of surviving under highly saline conditions, but actually benefit from the presence of substantial quantities of salt in the growth media [2,3]. For most of these species, optimal growth is achieved at salinity levels ranging between 150 and 200 mM (e.g., *Salicornia* [4]; *Atriplex* [5]; quinoa [6]), while for others, the optimum salt level in the media can be as high as seawater, *i.e.*, ~500 mM NaCl (*Sarcocornia fruticose* and *Arthrocnemum macrostachyum*; [7]). This is in stark contrast to glycophytes; most wheat varieties will show little if any yield at 150–200 mM NaCl and not a single rice genotype will be able to survive under such salinity levels [8,9]. So, how do these halophyte species flourish under saline conditions and how can we make wheat and rice as tolerant as halophytes?

It is generally accepted that the hallmark of salinity tolerance in halophytes is efficient vacuolar Na^+^ sequestration [10,11]. As such, halophytes are classified as Na^+^ includers, while most crops adopt a Na^+^ exclusion strategy [12,13]. However, as commented by many authors [3,11], there is no a clear-cut line between halophytes and glycophytes. Salt tolerance in plants is not an either or condition; it represents a continuum of degrees of tolerance to salinity. Salt-tolerant glycophyte species, such as barley, have successfully implemented key physiological mechanisms that confer salinity tolerances in halophytes. These include high tissue tolerance, efficient control of xylem Na^+^ loading and the use of inorganic ions for osmotic adjustment [14–16]. Indeed, it appears that there is nothing unique to halophytes that is not present in glycophytes. The major difference is how efficiently these mechanisms are implemented by the two plant groups [3].

Over the last few years, the research focus of our laboratories has been on quinoa (*Chenopodium quinoa* Willd.), a C3 facultative halophyte species of high nutritional and agronomical value [17,18]. Despite showing remarkable salinity tolerance [6,19], quinoa genotypes nevertheless display significant variability in agronomical and physiological responses when grown under saline conditions [20]. The physiological basis for this genetic variability in salinity tolerance in quinoa, as well as in other halophytic species, is not fully understood. Our very recent study involving 14 quinoa varieties revealed that, despite all being halophytes, quinoa genotypes are clustered into two distinct groups—“includers” and “excluders”—according to their ability to accumulate Na^+^ in the shoot [21]. Previously, such opposite strategies have been described mainly for highly contrasting species (e.g., wheat and barley) or when comparing adaptive mechanisms between glycophytes and halophytes [11]. Now, it appears that even within the same species of a halophyte, multiple strategies are used to deal with salinity. The reasons for this are unclear, as are the mechanisms involved. Can this duality in strategies of handling Na^+^ accumulation in the shoot be related to differential tissue tolerance among quinoa varieties or is it associated with differences in the ability to effectively sequester Na^+^ to the vacuole?

Efficient vacuolar sequestration of cytotoxic Na^+^ has always been considered as one of the most, if not the most, prominent feature of halophytes [10,11,22]. This process requires two complementary components: (1) active Na^+^ pumping into the vacuole against the electrochemical gradient; and (2) preventing Na^+^ from leaking back into cytosol [23]. While the molecular basis of the first component is well defined and is attributed to activity of tonoplast Na^+^/H^+^ antiporters [22,24], the mechanisms responsible for preventing Na^+^ from leaking back into the cytosol remain elusive. Recently, we showed that the properties of Na^+^-permeable fast- (FV) and slow- (SV) vacuolar channels differed dramatically between young and old quinoa leaves grown under saline conditions [25]. The SV channel is permeable to both mono- and di-valent cations and is activated by cytosolic Ca^2+^ and positive vacuolar voltage, while the FV channel is permeable for monovalent cations only and is inhibited by divalent cations ([25] and references within). We showed that at physiologically relevant tonoplast potentials that favour Na^+^ leak from the vacuole (e.g., 0 to 20 mV), most FV channels were functionally inactive in salt-grown old leaves, while FV conductance in young leaves grown under similar conditions was at least two-fold higher. This mirrors the amount of Na^+^ accumulated in mesophyll cells. Also, the number of active SV channels in young leaves (containing less Na^+^) exceeded the number for old leaves by seven-fold under saline conditions. Most of the SV channels were closed at physiologically relevant tonoplast potentials in salt-grown old leaves, while in young leaves, SV currents were substantial [25]. It was suggested that quinoa plants are able to control the activity of SV and FV tonoplast channels to match the specific growth conditions by ensuring that most of accumulated Na^+^ is safely locked in the vacuole of old leaves.

This work extends the above findings by comparing the properties of tonoplast FV and SV channels in two quinoa genotypes contrasting in their salinity tolerance. The results are complemented by studies of the kinetics of net ion fluxes across the plasma membrane of quinoa leaf mesophyll tissue. Taken together, our results suggest that multiple and highly orchestrated ionic mechanisms contribute to salinity tolerance in quinoa and this determines the genotypic variability in this trait in the *Chenopodium* family.

## 2. Results

**Figure 1:**
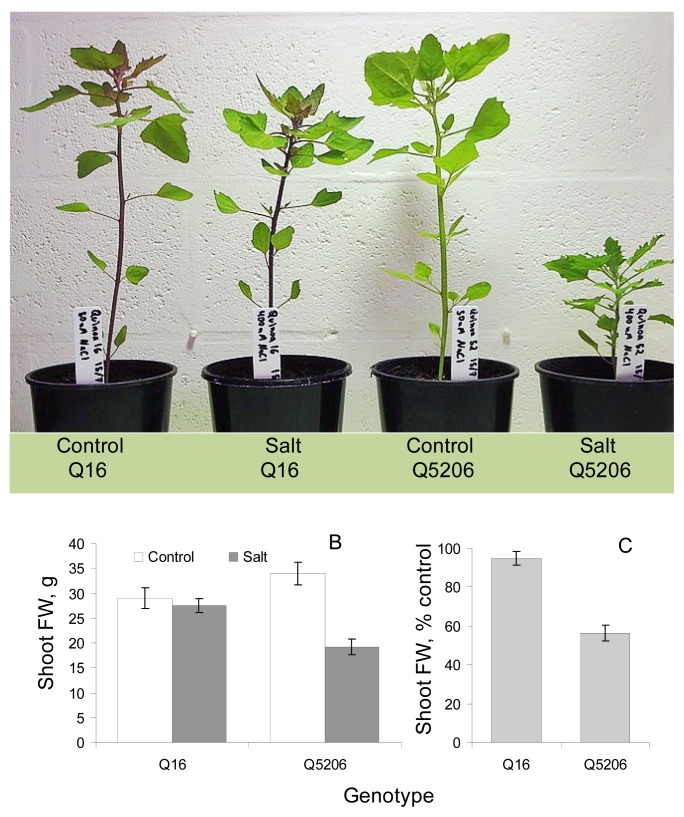
Genotypic differences in salinity stress tolerance in quinoa. Plants were grown under optimal (50 mM NaCl; defined as control) and saline (400 mM NaCl) conditions for four weeks. (**A**) Visual difference in genotypic responses to salinity; (**B**) shoot fresh weight (FW) of quinoa plants grown under control and saline conditions; (**C**) shoot FW of salt-grown plants expressed as % of control. Mean ± SE (*n* = 6). Data labelled with asterisk are significant at *p* < 0.05.

Four weeks of 400 mM NaCl treatment significantly reduced growth in the sensitive genotype Q5206, but had no significant (at *p* < 0.05) impact on the performance in the tolerant genotype Q16 (Figure 1). Salt-grown Q5206 plants looked stunted (Figure 1A) and their biomass was only at ~50% of the control (Figure 1B,C). No such reduction was found in the Q16 genotype (Figure 1). These results are therefore consistent with our previous findings that report a superior salinity tolerance in Q16 [20,21], paving the foundation for their use as contrasting genetic material in this work.

**Figure 2:**
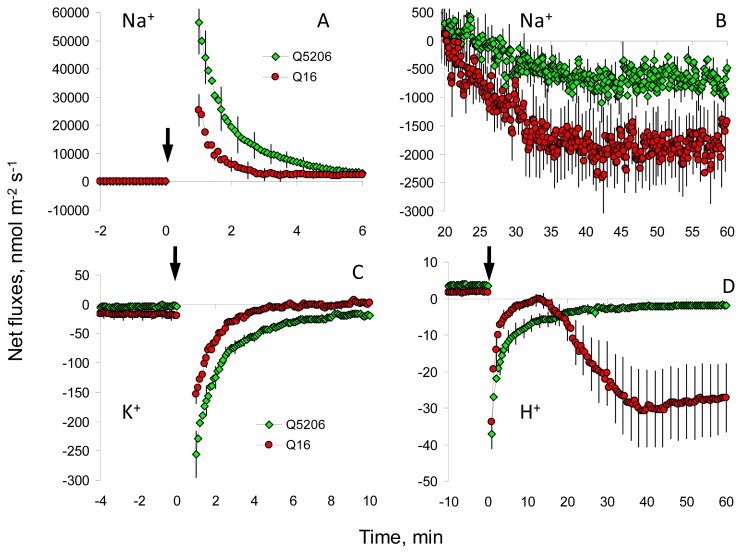
Net ion flux kinetics measured from leaf mesophyll tissue in response to acute 100 mM NaCl treatment (added at the time indicated by an arrow). Two quinoa varieties contrasting in their salinity tolerance (Q16, tolerant; Q5206, sensitive) were used. (**A**,**B**) net Na^+^ flux resolved at initial (first 5 min) and later (1 h after stress onset) stages of stress response. (**C**,**D**) net K^+^ and H^+^ fluxes, respectively. Mean ± SE (*n* = 6–8). The sign convention is “influx positive”.

Prolonged salinity treatments can result in substantially raised xylem Na^+^ contents, typically reaching a range of 50 to 100 mM (reviewed in [3]). The accumulation of such high Na^+^ concentrations in the leaf apoplast may affect mesophyll cell ionic homeostasis, with major implications for metabolic performance. This issue was addressed in this work by measuring the kinetics of net ion flux responses to acute salinity treatment (100 mM NaCl added directly to the leaf mesophyll; Figure 2). A significant genotypic difference was found in net ion flux responses of quinoa mesophyll to salinity for all the measured ions.

Acute salt stress caused an immediate uptake of Na^+^ into the leaf mesophyll; an uptake that was two-fold higher in the salt-sensitive Q5206 genotype than the tolerant genotype Q16 (significant at *p* < 0.05; Figure 2A). This net Na^+^ uptake was short lived, and 20 min after stress onset, both genotypes switched to net Na^+^ efflux (pumping Na^+^ out; Figure 2B). The rate of Na^+^ pumping was three-fold higher in the tolerant genotype Q16 (Figure 2B; significant at *p* < 0.05). Two major propositions can be made based on these observations: (i) an active Na^+^ efflux system is present at the plasma membrane of leaf mesophyll cells (e.g., a functional homologue of the *Arabidopsis* SOS1 Na^+^/H^+^ exchanger; [26]) and (ii) the activity of this active efflux system correlates with salinity tolerance in quinoa.

Similar to our previous observation from other species [27–29], salinity treatment resulted in a massive K^+^ leak from the leaf mesophyll (Figure 2C). In the tolerant genotype Q16, this leak was much less (~two-fold) than in the sensitive Q5206 genotype (significant at *p* < 0.05). Taken together, the better K^+^ retention (Figure 2C) and better Na^+^ exclusion (Figure 2B) ability of the genotype Q16 would enable a more optimal cytosolic K^+^/Na^+^ in the cytosol of this genotype.

In other species, such as barley [14] and lucerne [30], a better K^+^ retention ability in tolerant varieties is always attributed to the maintenance of a more negative membrane potential under saline conditions; a trait conferred by higher H^+^-ATPase activity [31]. Here, we report indirect evidence that differential H^+^-ATPase activity may also be the case for the observed genotypic differences in salinity stress tolerance in quinoa. Indeed, while in the sensitive Q5206 variety, NaCl-induced stimulation of H^+^ efflux was transient and lasted for only a few minutes; in the tolerant Q16 genotype, a massive H^+^ efflux was measured 1 h after stress onset. Similar to reports on other species [27], this NaCl-induced H^+^ efflux was vanadate-sensitive (data not shown), suggesting that it is mediated by the plasma membrane H^+^-ATPase. This H^+^ efflux would be expected to restore (an otherwise depolarised) cell membrane potential, which would explain the better K^+^ retention in Q16 (Figure 2C), assuming that, similar to many other species [32], K^+^ leak in quinoa is mediated by depolarisation-activated outward-rectifying K^+^ channels (GORK in *Arabidopsis*; [33]).

**Figure 3:**
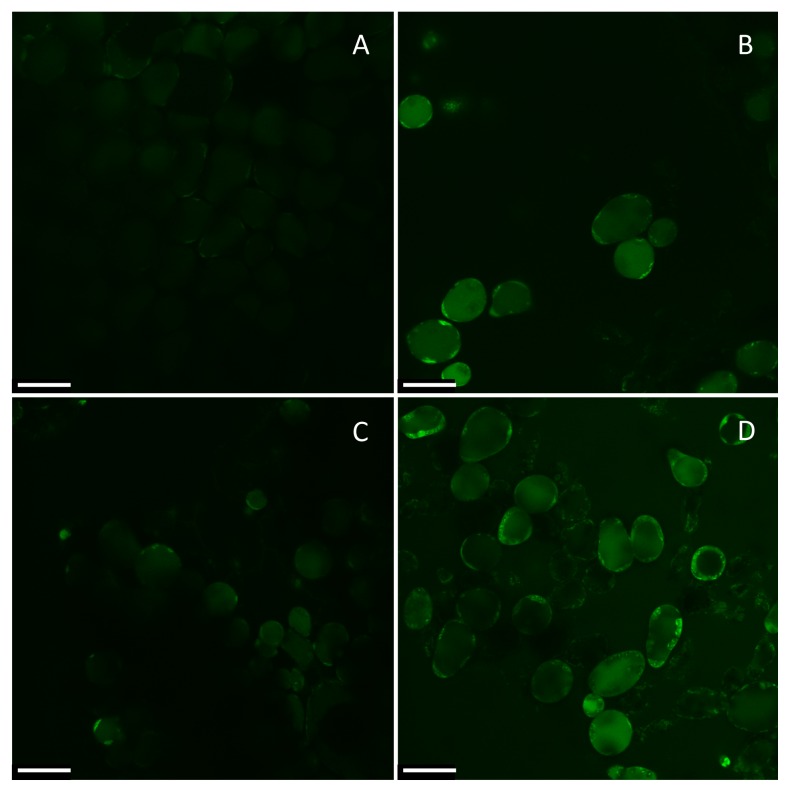

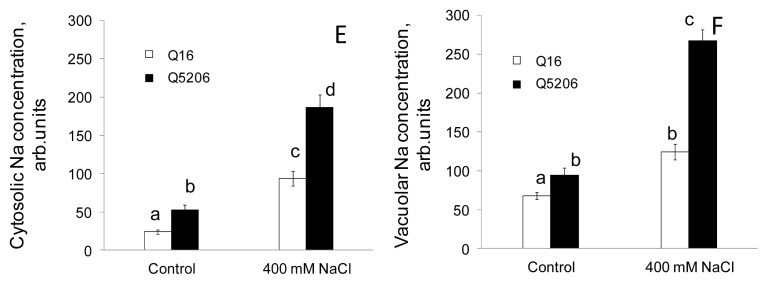
Na^+^ accumulation in quinoa leaves, visualised by confocal imaging using CoroNa Green fluorescent dye. One (of six to 10) representative images is shown for each treatment. The scale bar size is 50 μm. (**A**) Q16 leaf in control; (**B**) Q16 leaf grown at 400 mM NaCl; (**C**) Q5206 leaf in control; (**D**) Q5206 leaf at 400 mM NaCl. Plants grown under saline conditions accumulate more Na^+^ in leaf mesophyll cells, as evident by more intensive green colour. (**E**,**F**) cytosolic (E) and vacuolar (F) Na^+^ concentrations in quinoa mesophyll cells quantified from confocal CoroNa Green data (arbitrary units). Mean ± SE (*n* = 40). Different lowercase letters indicate the significance level at *p* < 0.05.

The predicted genotypic difference in cytosolic Na^+^ accumulation based on MIFE Na^+^ flux data (Figure 2A,B) was further investigated *in planta* using CoroNa Green fluorescent dye (Figure 3). This revealed that cytosolic Na^+^ levels were substantially lower in the tolerant Q16 genotype (two-fold; significant at *p* < 0.05) (Figure 3E). The same two-fold difference was also found for vacuolar Na^+^ concentrations (Figure 3F). These observations are consistent with our previous reports of Q16 being an “excluder”, accumulating less Na^+^ in the shoot compared to Q5206 [21].

As noted in the Introduction, efficient control of tonoplast FV and SV channels is essential for vacuolar Na^+^ sequestration. We therefore compared the properties of FV and SV currents in isolated mesophyll vacuoles of two quinoa varieties contrasting in their salinity tolerance.

**Figure 4:**
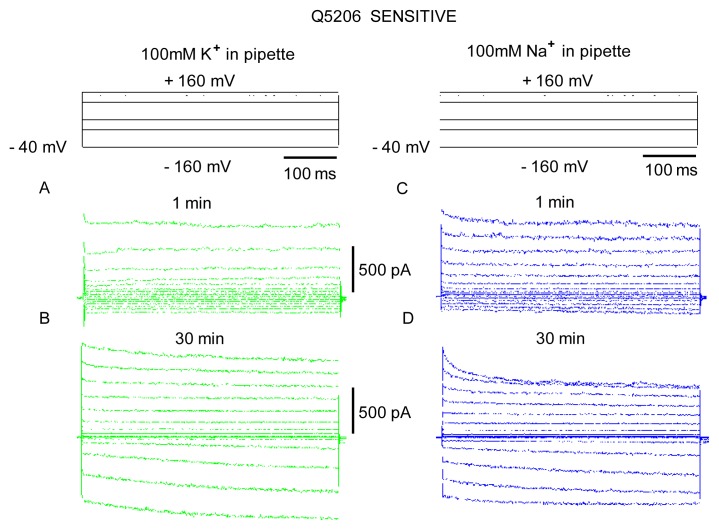
Typical whole vacuole recordings of fast-activating vacuolar (FV) currents from the Q5206 genotype, illustrating a run-up phenomenon in patch-clamp measurements. (**A**,**B**) typical FV current recordings in a symmetrical 100 mM KCl solution from small (C ~3 pF) quinoa tonoplast vesicles, isolated from a large central vacuole, shortly (1 min; defined as initial; panel **A**) and 30 min after obtaining a whole vacuole configuration (defined as final; panel **B**); (**C**,**D**) as above, for 100 mM NaCl in the pipette. One (of six to 10 vacuoles) typical recording is shown for each case.

Tonoplast conductance is dominated by instantaneously activated FV currents at zero free concentrations of divalent cations (Ca^2+^ and Mg^2+^) on both sides of the membrane. Recordings immediately after breaking into the whole vacuole configuration revealed instantaneous currents with a marked outward rectification (larger currents evoked by cytosol-positive voltages, Figure 4). This current remained stable over a period of time (typically up to 15–20 min), before suddenly growing to reach a new stable state (illustrated by comparing panels A and B). This phenomenon is known as a run-up and has previously been described for FV currents in other plant systems [34]. Regardless of the nature of the cation used in a pipette (e.g., K^+^*vs.* Na^+^), FV currents were about the same (Figure 4) and showed the same run-up patterns. This is consistent with previous reports of FV channels being almost equally selective for Na^+^ and K^+^ [25,35] and suggests that either ion can be used to study the properties of FV channels in quinoa.

**Figure 5:**
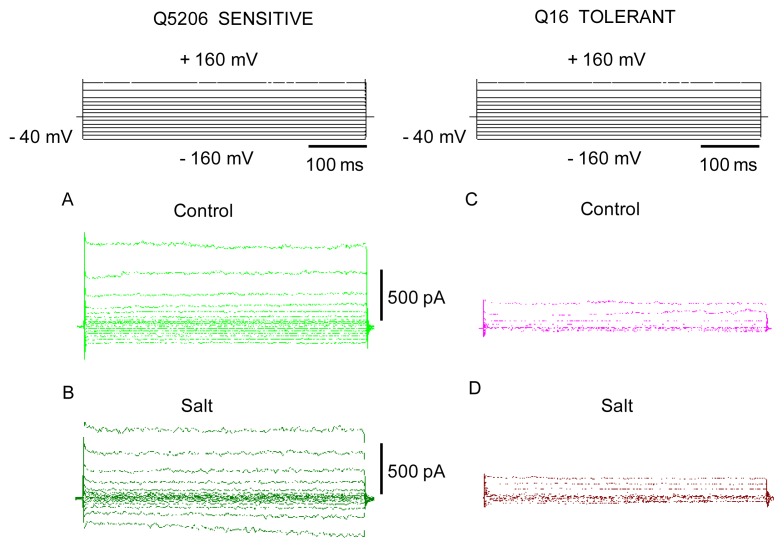
A comparison of the magnitude of the initial fast-activating (FV) currents, measured in the whole vacuole mode in salt-tolerant Q16 and salt-sensitive Q5206 quinoa genotypes grown under control (**A**,**C**) and high (400 mM) salt (**B**,**D**) conditions. One typical recording is shown for each case (five to eight vacuoles for each genotype). Whole vacuole capacitance was ~3 pF in all cases.

When the initial FV currents were compared in the two varieties, the current density was substantially smaller in the tolerant Q16 genotype (Figure 5A,C, respectively). No noticeable effects of the growth conditions (e.g., control *vs.* salt-grown) were found in either genotype (Figure 5A–D, respectively).

**Figure 6:**
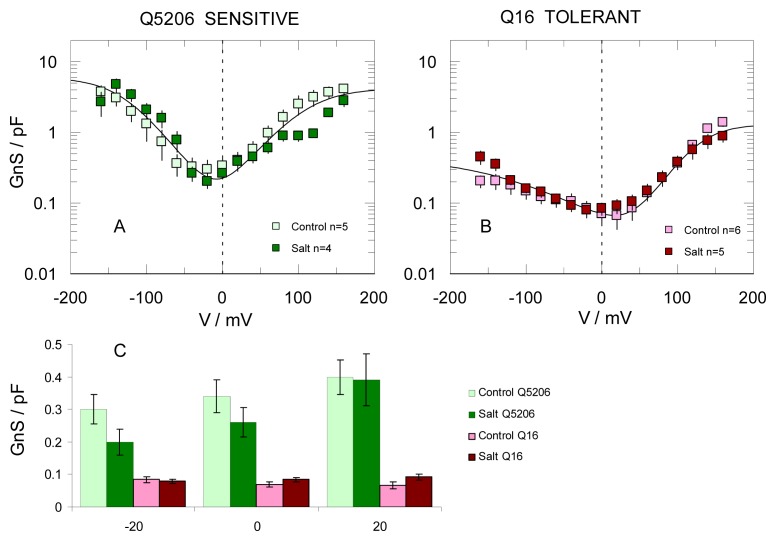
FV currents are smaller in vacuoles isolated from the salt tolerant Q16 genotype. (**A**,**B**) Whole vacuolar FV conductance was evaluated in salt-sensitive (Q5206; panel **A**) and salt-tolerant (Q16; panel **B**) genotypes by taking the first derivative of the whole vacuole I/V relationship, measured immediately after achieving the whole vacuole configuration. Vacuoles were isolated from leaves of quinoa plants grown in the presence of 50 mM NaCl (control) or saline (400 mM NaCl for four weeks, salt). Data are means ± SE. Solid lines are best fits to a three-state (“*open1-closed-open2*”) model [36]. Pipette and bath solutions contained 100 mM KCl; (**C**) Same data as **A**,**B**, but only the points within the physiological tonoplast potential range (±20 mV) are considered.

The above observations are further supported by the statistical information reported in Figure 6. A semi-logarithmic plot of conductance *vs.* voltage (G/V) was used to visualise FV channel voltage-dependent activity (Figure 6A,B). Of specific interest are FV conductances at physiological (near-zero) tonoplast potentials, presented separately in Figure 6C. Within this range (−20 to +20 mV), FV conductance in the salt-sensitive Q5206 genotype was three- to five-fold higher than the salt-tolerant Q16. Nonetheless, the growth conditions had no significant (*p* < 0.05) observable effect (open and closed symbols in Figure 6A,B).

**Figure 7:**
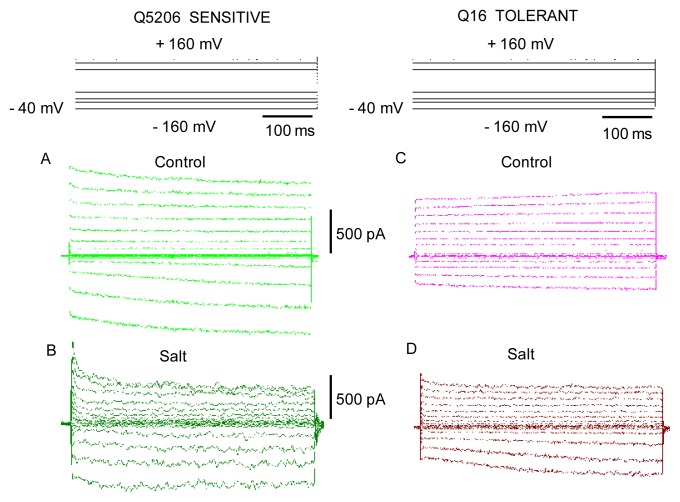
A comparison of the amplitude of the final steady-state whole-vacuole fast-activating (FV) currents between salt tolerant Q16 and salt-sensitive Q5206 genotypes, grown under control (**A**,**C**) and high (400 mM) salt (**B**,**D**) conditions. One typical recording is shown for each case (obtained from five to eight vacuoles). Vacuolar vesicles with a capacitance ~3 pF were chosen for this illustration.

**Figure 8:**
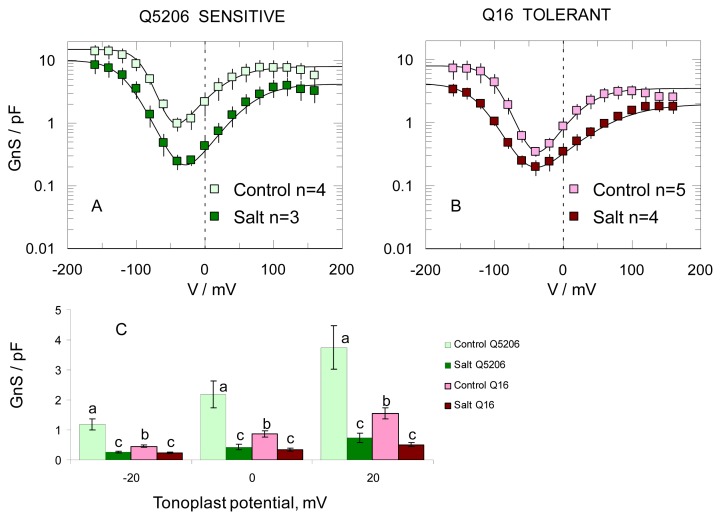
Salinity reduces the final vacuolar FV currents in quinoa. (**A**,**B**) Whole vacuolar FV conductance was evaluated in salt-sensitive (Q5206; panel **A**) and salt-tolerant (Q16; panel **B**) varieties by taking the first derivative of the whole vacuole I/V relationship, measured 30 min after achieving the whole vacuole configuration. Growth conditions, pipette ion composition and data analysis are the same as in Figure 6; (**C**) Same data as **A**,**B**, but only the points within the physiological tonoplast potential range (±20 mV) are shown.

The effect of the growth conditions was very pronounced though when FV currents were analysed in their final state (e.g., 30 min after vacuole perfusion and seal formation). Salt grown plants (treated with 400 mM NaCl for four weeks) showed greatly reduced FV currents (Figure 7). While this phenomenon was observed in both genotypes (Figure 7), it was more pronounced in the salt-sensitive Q5206 genotype (4.9 ± 0.2-fold in Q5206 *vs.* 2.5 ± 0.3-fold in Q16, respectively, within the physiologically relevant range of tonoplast potentials; Figure 8).

In the final state, the I/V curve of the FV current was N-shaped, presenting a high resistance (low conductance) region of *ca*. −40 mV under symmetrical 100 mM KCl conditions. In contrast, at high potentials of either sign, the conductance steeply increased (Figure 8A,B). Vacuolar FV currents were 2–3-fold higher in the salt-sensitive Q5206 genotype than in the salt-tolerant Q16, within a physiologically relevant range of tonoplast potentials (Figure 8C).

Another possible pathway mediating Na^+^ leak into the cytosol could be via SV channels [37]. In quinoa, these channels were shown to be slightly more permeable for Na^+^ than K^+^ (P_Na/K_ = 1.6–1.8). Keeping in mind that high vacuolar Na^+^ decreases the threshold for SV channel voltage activation [38], an even tighter control of SV channels is required under saline conditions to avoid their mediation of Na^+^ leakage.

**Figure 9:**
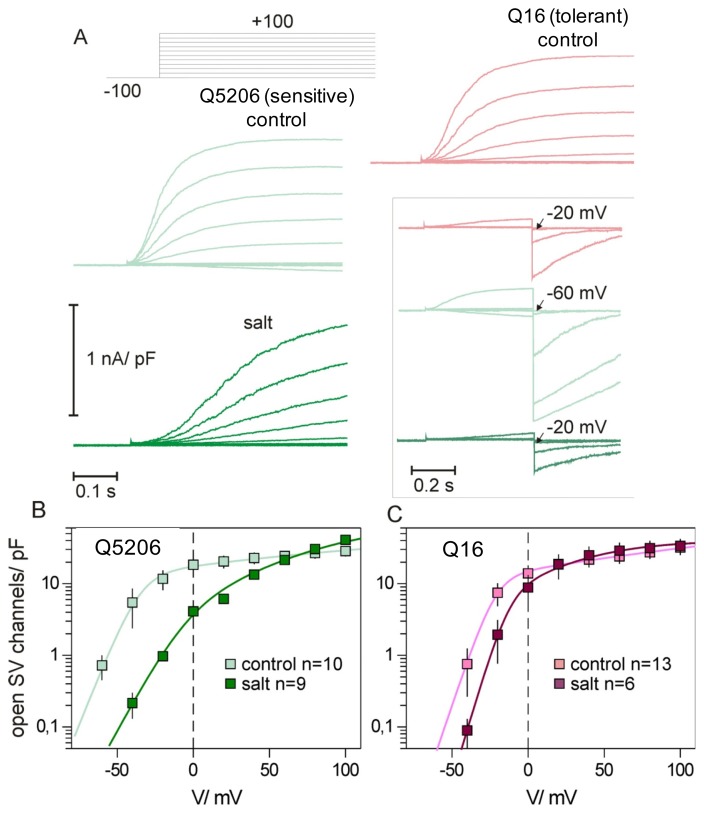
Effects of salinity on the activity of SV channels in mesophyll vacuoles from two contrasting quinoa genotypes. (**A**) Typical records of the SV channel activation in response to positive voltage steps. Inset (same colour coding) shows parts of the same recordings (only the traces between −60 to +20 mV) followed by a step to −100 mV (deactivation of the SV; the so-called tail currents). The arrow indicates the threshold for the SV channel voltage activation (*i.e.*, first test voltage, after which some tail current was observed); (**B**,**C**) Activation curves for salt-sensitive Q5206 and salt-tolerant Q16 varieties. Mean number of open SV channels (calculated as a tail current at −100 mV, divided by the single channel current at this potential) at tonoplast unit surface unit (1 pF~100 μm^2^) is shown as a function of test voltage. Solid lines are the best fits to the pair of Boltzmann distributions. Data are mean ± SE (*n* = 6–13). Plants were grown either under control (50 mM NaCl) or saline (400 mM NaCl) conditions. Recordings were made under symmetric 100 mM KCl conditions. One millimolar Ca^2+^ was added to the cytosolic side to activate SV and inhibit FV currents.

Figure 9 compares the activity of SV channels in the mesophyll tonoplast of salt-sensitive (Q5206) and salt-tolerant (Q16) quinoa varieties. The original records (Figure 9A) show that in the salt-sensitive genotype, the threshold for the activation is quite low (at −60 mV). However, it switches to −20 mV in salt-grown plants, these also displaying a slower SV current activation. A shift of voltage dependence, induced by salt, implies a 10-fold decrease of mean activity of SV channels at physiologically attainable transtonoplast potentials [(−20)–0 mV, Figure 9B]. Regarding the salt-tolerant Q16, under control conditions, the SV activation curve for this genotype already resembles that for salinized Q5206 plants, while for salt-grown Q16 plants, only a moderate shift of the threshold for the SV voltage activation was observed (Figure 9B).

## 3. Discussion

Salinity tolerance in plants is a complex trait, both physiologically and genetically [1]. Multiple mechanisms are involved, and their relative contribution may differ dramatically depending on the developmental stage of the plant, severity of the salinity stress and any confounding effects of other environmental factors. Indeed, while a plant’s ability to control stomata to maintain the optimal balance between water availability and leaf transpiration rate in saline soils may be critical for advanced plants [39], this trait is of no importance at germination or the coleoptile stage (when stomatal transpiration is almost non-existent). Another example is Na^+^ exclusion from uptake. In plant roots, this exclusion is mediated by a SOS1-like Na^+^/H^+^ exchanger [26], fuelled by the plasma membrane H^+^-ATPase. If salinity problems coincides with flooding (a situation often found in nature; [40]), the root’s ability to exclude Na^+^ will be compromised by the lack of oxygen required to maintain a high H^+^-ATPase activity. Under these conditions, tissue tolerance mechanisms, such as ROS scavenging or vacuolar Na^+^ sequestration, may become much more important. Consequently, we should accept the fact that there is no such thing as a “silver bullet” that can resolve salinity problems; manipulating only one trait (gene) is not likely to result in any significant improvements. Salinity tolerance problems will only be resolved when several key traits are combined in a complementary manner. Nonetheless, the introgression of the single Nax2 gene has been shown to improve the grain yield in durum wheat by 20% under saline conditions [41], suggesting that some traits are of greater importance than others are. The nature of these traits may be learned from halophytes, by definition, the most salt tolerant species.

Our previous studies on quinoa have identified several whole-plant physiological traits that could be essential for the high salinity tolerance in this species [6,18,20,21,42]. Here, we extend this list by adding several intracellular mechanisms that contribute to the genetic variability in salinity tolerance. These mechanisms include: (i) a higher rate of Na^+^ exclusion from the leaf mesophyll; (ii) maintenance of low cytosolic Na^+^ levels; (iii) better K^+^ retention in the leaf mesophyll; (iv) more active H^+^ pumping, enabling the restoration of membrane potentials in mesophyll cells; and (v) an ability to reduce the activity of SV and FV channels under saline conditions (Table 1).

These traits, which differ between sensitive and tolerant quinoa genotypes, are well orchestrated, forming several “lines of defence” in mesophyll cells. First, the tolerant genotype has a smaller net Na^+^ uptake into the mesophyll (Figure 2A) and has a superior capacity to extrude accumulated Na^+^ (Figure 2B). We have previously shown that in wheat and *Arabidopsis*, such a net Na^+^ efflux is attributed to the activity of SOS1-like proteins in the plant root epidermis [43]. We suggest that a similar scenario occurs in the quinoa mesophyll. Indeed, functional *SOS1* analogues have been reported in quinoa [44,45], and saline treatment (450 mM NaCl) has been shown to cause an up-regulation of *SOS1* in quinoa leaves [44].

At the same time, the tolerant quinoa genotype had much a better ability to retain K^+^ in the leaf mesophyll (Figure 2C). Maintenance of a high cytosolic K^+^ is not only important for the activity of a large number of cytosolic enzymes [46], but is also essential for suppressing the activity of caspase-like proteases and endonucleases in plant cells [47–49]. If depletion in the cytosolic K^+^ pool cannot be prevented, protein catabolism takes place, resulting in programmed cell death (PCD) [48,50]. In *Arabidopsis* and barley, depolarisation-activating outward rectifying K^+^ channels are the main route for K^+^ efflux under saline conditions [32]. As such, restoring membrane potential by more active H^+^ pumping is a possible way of controlling such a leak, so preventing stress-induced PCD. This seems to be the case for the quinoa mesophyll. A sustained net H^+^ efflux was induced in the salt-tolerant Q16 genotype by NaCl treatment (Figure 2D), while such activation was very short-lived in the sensitive Q5206.

Together, better Na^+^ exclusion to the apoplast (Figure 2B) and a higher K^+^ retention in the cytosol (Figure 2C) will ensure a more optimal cytosolic K^+^/Na^+^ ratio, a key feature conferring salinity tolerance in plants [51].

The tolerant Q16 genotype was capable of maintaining much lower cytosolic Na^+^ levels (Figure 3). Two major mechanisms contributed to this feature. First, as discussed above, the Q16 genotype had much better ability to actively extrude Na^+^ (Figure 2B). Second, the activities of the Na^+^-permeable tonoplast SV and FV channels were much lower in the tolerant genotype (Figures 5 and 9). Earlier, we showed [25] that quinoa’s tonoplast SV and FV activity is strongly affected by the amount of Na^+^ accumulated in leaf tissue. Both FV and SV channel activity were several fold lower in old leaves (accumulating more Na^+^) compared to young ones. This reduction was interpreted as a need to prevent Na^+^ leaking back into the cytosol after its sequestration to the vacuole by a NHX-like Na^+^/H^+^ exchanger [22]. Thus, it appears that this ability to control tonoplast channel activity is not only stress-inducible, but is also genetically controlled and contributes to the differential salinity tolerance between genotypes.

Interestingly, vacuolar Na^+^ concentrations were higher in the sensitive Q5206 than in the tolerant Q16 genotype, both in control and in salt-treated plants (Figure 3). This may be due to a better ability to control Na^+^ delivery to the shoot in the Q16 genotype, so the plant has less Na^+^ in the shoot. Nonetheless, both genotypes are classified as halophytes, so can sequester substantial amounts of Na^+^ in their vacuoles. In this context, the higher Na^+^ sequestration ability of Q5206 may be a compensatory trait accounting for its poorer Na^+^ exclusion ability.

One more result should be commented upon. Both FV (Figure 8) and SV (Figure 9) channel activity was significantly reduced in plants grown under high (400 mM NaCl) salinity. It appears that in a tolerant genotype, the tonoplast channel activity is intrinsically lower, while in sensitive genotypes, this reduction is stress-inducible. This pattern is reminiscent of that related to the expression of tonoplast NHX Na^+^/H^+^ antiporters. These antiporters are constitutive in halophytes [10,24,52], but are induced by NaCl in glycophytes [53–55]. Thus, any difference between plant performance will be maximal at the onset of salt stress, because stress-inducible down-regulation of FV and SV channels in salt-sensitive genotypes may take some time. It remains to be answered as to whether these changes in SV and FV activity originate from changes at the transcript level (expression patterns) of these transporters under saline conditions, post-translational regulation or the existence of alternative splicing forms or whether the observed difference is attributed to channel regulation by some cytosolic or luminal compound(s). The latter seems to be true for FV channels. These are likely to be controlled by some, as yet unknown, luminal factor, which can be washed out from the vacuole. This would reduce the difference in FV activity between the salt-sensitive and salt-tolerant varieties (Figures 6 and 8). The nature of this factor, which controls the run-up of the whole vacuole FV activity under patch-clamp conditions, as well as of those that control the threshold for SV channels activation under salt stress, has to be addressed in future experiments.

## 4. Experimental Section

### 4.1. Plant Material and Growth Conditions

Two quinoa (*Chenopodium quinoa* Willd.) varieties contrasting in salinity tolerance [18,21] were used in this study. A relatively salt-sensitive variety Q5206 (also known as Titicaca) is an early maturing, day-length neutral cultivar that has been bred and selected for North European conditions at the Faculty of Sciences, Univ. Copenhagen, Denmark, from material originating from crosses between southern coastal Chilean and Peruvian altiplano lines, followed by the mass selection. A more salt-tolerant variety Q16 (also known as Huallata) is a traditional short day cultivar of the real type from the southern Bolivian Altiplano (20°28′S, 66°50′W, 3650 m above sea level). Plants were grown from seed under controlled greenhouse conditions (temperature between 19 and 26 °C; day length, 12 h; average humidity ~65%) at the University of Tasmania between April 2012 and November 2012. Plants were grown in 2 L plastic pots using a standard potting mix (70% composted pine bark; 20% coarse sand; 10% sphagnum peat; Limil at 1.8 kg/m^3^, dolomite at 1.8 kg/m^3^). The plant nutrient balance was maintained by adding the slow release Osmocote Plus™ fertilizer (at 6 kg/m^3^), plus ferrous sulphate (at 500 g/m^3^). Once plants were 3-weeks old, salinity stress was given using 400 mM NaCl for irrigation. Control plants were irrigated with 50 mM NaCl (a concentration found to be stimulating for quinoa growth). Salt treatment was provided as 40 mM/day increments. The final concentration of 400 mM was reached at day 10 and maintained for a further seven weeks.

### 4.2. Vacuole Isolation and Patch-Clamp Electrophysiology

Vacuoles were mechanically isolated from mesophyll protoplasts obtained by enzymatic digestion as described in our recent work [25]. The enzyme solution contained 2% (*w*/*v*) cellulose (Yakult Honsha, Tokyo, Japan), 1.2% (*w*/*v*) cellulysin (Biosciences Inc., San Diego, CA, USA), 0.1% (*w*/*v*) pectolyase, 0.1% (*w*/*v*) bovine serum albumin, 10 mM KCl, 10 mM CaCl_2_ and 2 mM MgCl_2_, pH 5.7, adjusted with 2 mM MES. Solution osmolality was adjusted to 750–800 mOsm for control and to 1700–1800 mOsm for salt-grown plants using sorbitol. Once released by plasmolysis, protoplasts were washed and kept in a “storage solution” (100 mM KCl; 0.5 mM EDTA; 10 mM HEPES; 10 mM glucose; 10 mM sucrose; pH 7.4; osmolality 500–570 mOsm for control or 1450–1500 mOsm for salt-grown leaves) on ice. Current measurements were performed using an Axopatch 200B Integrating Patch-clamp amplifier (Axon Instruments, Foster City, CA, USA), as described elsewhere [25]. The standard pipette solution contained (in mM): 100 KCl, 5 EGTA and 10 MES-KOH (pH 6). Bath solution for FV currents contained (in mM): 100 KCl, 0.5 EDTA, 10 HEPES-KOH (pH 7.4). To inhibit FV and activate SV currents, 0.5 EDTA was substituted with 1 CaCl_2_. Specific voltage protocols are given directly on the figures.

### 4.3. Intracellular Na^+^ Measurements

The cytosolic and vacuolar Na^+^ content was measured using fluorescent Na^+^ dye (CoroNa Green acetoxymethyl ester; Invitrogen, Carlsbad, CA, USA), as described in our previous work [25]. Quinoa plants were grown in a glasshouse, as described above. The youngest fully expanded leaves were collected; abaxial epidermis was peeled off with a pair of fine forceps and peeled leaf segments were incubated in a buffer solution containing CoroNa Green in the dark for 1 h before measurement. Confocal imaging was conducted on a Leica inverted microscope fitted with a TCS SPII confocal head (Leica Microsystems, Heidelberg, Germany), as described in [25]. Images were analysed with LAS AF software (Leica Microsystems, Heidelberg, Germany) and Image J software (NIH, USA) to calculate vacuole or cytosol fluorescence corrected for the background [56]. Results are reported per surface area. This represents Na^+^ concentrations in an appropriate compartment expressed in arbitrary units.

### 4.4. Non-Invasive Ion Flux Measurements

Net fluxes of K^+^, Na^+^ and H^+^ were measured using non-invasive microelectrode ion flux estimation (the MIFE) technique (Univ. Tasmania, Hobart, Tasmania), essentially as described in our previous publications [14,27,42,57]. Commercially available K^+^ (catalogue No 60031) and H^+^ (catalogue No 95297) LIX were used (both from Sigma-Aldrich, St. Luis, MO, USA). For Na^+^ measurements, an improved calixarene-based Na^+^ cocktail was used (see [58] for details). The principle of the MIFE measurements and all details on microelectrode fabrication and calibration are available in previous publications (e.g., [57]). In brief, glass microelectrodes were pulled from non-filamentous glass capillaries, salinized with tributylchlorosilane (Fluka Chemicals 90796, Busch, Switzerland) and filled with the appropriate ion-selective cocktail. Electrodes were calibrated in a set of pH, K^+^ or Na^+^ standards, then used for measurements. One youngest, but fully mature leaf was harvested and brought into laboratory in a sealed plastic bag. The abaxial leaf epidermis was removed using fine tweezers, and leaf segments of approximately 5 × 8 mm were cut and left floating (peeled side down) overnight in the Basic Salt Medium (BSM) containing 0.5 mM KCl, 0.1 mM CaCl_2_, pH 6.0 (not buffered). To eliminate possible confounding wounding effects, measurements were conducted next day (see [27] for all methodological aspects). One hour prior to measurement, leaf segments were immobilised in the measuring chamber and electrode tips were positioned 30 μm above the leaf surface, with their tips aligned and separated by 1–3 μm. During measurement, electrodes were moved back and forward in a square-wave manner by a computerised stepper motor between two positions, 80 and 30 μm above the leaf surface, with 0.1 Hz frequency. Net ion fluxes were calculated from the measured differences in electrochemical potential for each ion between these two positions as described elsewhere [57].

## 5. Conclusions

Multiple mechanisms may contribute towards genotypic differences in salinity tolerance in quinoa. These include: (i) a higher rate of Na^+^ exclusion from the leaf mesophyll; (ii) maintenance of low cytosolic Na^+^ levels; (iii) a better K^+^ retention in the leaf mesophyll; (iv) a higher rate of H^+^ extrusion that contributes towards the restoration of the membrane potential in mesophyll cells; and (v) the ability to reduce the activity of SV and FV channels under saline conditions. These mechanisms appear to be highly orchestrated, thus enabling a remarkable overall salinity tolerance in quinoa species.

## Figures and Tables

**Table 1:** Intracellular traits differentiating salinity tolerance between quinoa genotypes.

Trait	Q5206 (sensitive)	Q16 (tolerant)
Na^+^ uptake into mesophyll	High	Low
Active Na^+^ exclusion	Low	High
Cytosolic Na^+^ level	High	Low
K^+^ retention in leaf mesophyll	Low	High
Ability to restore MP	Low	High
FV activity a	High	Low
SV density b	High	Low

a This reflects the FV activity in intact vacuoles, both in control plants and those subjected to salt stress. When the vacuolar lumen is perfused with an artificial pipette solution, the difference in the FV activity between them tends to decrease;

b This is true for plants grown under control conditions. Salt adaptation causes a shift in the threshold for SV activation in the salt-sensitive Q5206; its activity under these conditions approaches that of the salt-tolerant Q16.
